# Monitoring risk factors for dementia in middle-aged and older adults: a longitudinal study

**DOI:** 10.1590/1980-5764-DN-2023-0095

**Published:** 2024-04-22

**Authors:** Bruna Moretti Luchesi, Mariana Tiemi Kajiyama, Amanda Rocha Abreu, Marcelo Kwiatkoski, Tatiana Carvalho Reis Martins

**Affiliations:** 1 Universidade Federal do Mato Grosso do Sul, Campus Três Lagoas, Faculdade de Medicina, Três Lagoas MS, Brazil.; 2 Universidade Federal do Mato Grosso do Sul, Campus Três Lagoas, Programa de Graduação em Enfermagem, Três Lagoas MS, Brazil.

**Keywords:** Aged, Dementia, Middle Aged, Primary Prevention, Primary Health Care, Risk Factors, Idoso, Demência, Pessoa de Meia-Idade, Prevenção Primária, Atenção Primária à Saúde, Fatores de Risco

## Abstract

Given the importance of dementia syndrome and its impacts on the population, interest in studying modifiable risk factors for dementia is growing.

**Objective:**

To compare the prevalence of risk factors for dementia in middle-aged and older adults over a two-year period and to identify what variables in baseline were predictive of cognitive decline in the follow-up.

**Methods:**

Longitudinal and quantitative study, with follow-up evaluation after two years, conducted with 200 participants aged 45 years or more, registered in Primary Care Units. In the baseline (2018/2019) and follow-up (2021) assessments, sociodemographic data were collected, and cognitive performance and risk factors for dementia were evaluated (education, hearing loss, head trauma, high blood pressure, alcohol use, obesity, smoking, depressive symptoms, social isolation, physical inactivity, and diabetes mellitus). Data were compared using the McNemar’s test. Individual multinomial logistic regression models were performed to identify the factors associated with cognitive decline after two years.

**Results:**

The percentages of low education, traumatic brain injury, and smoking remained the same in both assessments. There was a significant increase in the prevalence of high blood pressure (from 55.0 to 62.0%) and physical inactivity (from 58.5 to 74.5%) and a significant reduction in social isolation (from 25.0 to 18.0%). Participants with depressive symptoms in baseline had a higher risk of cognitive decline in follow-up.

**Conclusion:**

There was an increase in the prevalence of high blood pressure and physical inactivity and a reduction in social isolation after two years. Depressive symptoms predict cognitive decline.

## RESUMO.

Dada a importância da síndrome demencial e de seus impactos na população, cresce o interesse em estudar os fatores de risco modificáveis para a demência.

**Objetivo:**

Comparar a prevalência de fatores de risco para demência em adultos de meia-idade e pessoas idosas, em um período de dois anos, e identificar quais fatores de risco na avaliação de base predizem o declínio cognitivo na avaliação de acompanhamento.

**Métodos:**

Estudo longitudinal e quantitativo, com avaliação de acompanhamento após dois anos, realizado com 200 participantes com 45 anos ou mais, cadastrados na Atenção Básica. Nas avaliações de base (2018/2019) e de acompanhamento (2021) foram coletados dados sociodemográficos, foram feitas avaliação cognitiva e avaliação dos fatores de risco para demência (escolaridade, perda auditiva, traumatismo craniano, hipertensão arterial, uso de álcool, obesidade, tabagismo, sintomas depressivos, isolamento social, inatividade física, diabetes *mellitus*). Os dados foram comparados pelo teste de McNemar. Modelos de regressão logística multinominal individuais foram conduzidos para identificar quais fatores estavam associados ao declínio cognitivo após dois anos.

**Resultados:**

Os percentuais de baixa escolaridade, traumatismo craniano e tabagismo mantiveram-se os mesmos nas duas avaliações. Houve aumento significativo da prevalência de hipertensão arterial (de 55,0 para 62,0%) e inatividade física (de 58,5 para 74,5%), e redução significativa do isolamento social (de 25,5 para 18,0%). Participantes com sintomas depressivos na avaliação de base apresentaram maior risco de declínio cognitivo no acompanhamento.

**Conclusão:**

Houve aumento das prevalências de hipertensão arterial e inatividade física; e redução do isolamento social. Sintomas depressivos predizem declínio cognitivo.

## INTRODUCTION

The number of people living with dementia has increased. Currently, it is estimated that the condition affects more than 55 million people worldwide, and this number is expected to grow to 78 million in 2030 and to 139 million in 2050^1^. However, only a quarter of the world’s countries have policies, plans, and strategies to support people with dementia and their families^1^.

Due to the importance of dementia syndrome and the relevance of its impacts on the population, there is growing interest in studying modifiable risk factors for cognitive impairment and dementia, especially in low- and middle-income countries, where the incidence of dementia is increasing faster^2^.

In 2017, a publication identified, through literature reviews and meta-analysis, the risk factors for dementia. It was estimated that 35% of cases could be avoided by eliminating potentially modifiable factors. The remaining 65% are related to genetic and unknown factors^3^. In 2020, after a review of the study, three new factors were identified, totaling 12, which are responsible for preventing or delaying 40% of dementia cases. The modifiable factors identified were divided into those that occur in young individuals (<45 years old), such as low education (7%); in middle aged (45-65 years old), such as hearing loss (8%), traumatic brain injury (TBI; 3%), high blood pressure (2%), alcohol use >21 units per week (1%), and obesity (1%); and in old age (>65 years), such as smoking (5%), depression (4%), social isolation (4%), physical inactivity (2%), air pollution (2%), and diabetes mellitus (1%)^4^.

Further investigations have been conducted in low- and middle-income countries to assess modifiable risk factors for dementia. A study conducted in India, China, and six Latin American countries identified that modifiable risk factors corresponded to 39.5%, 41.2%, and 55.8%, respectively^5^. In Mozambique, Brazil and Portugal, seven modifiable risk factors were evaluated, corresponding to 24.4%, 32.3% and 40.1% in each country, respectively^6^. If the prevalence of each factor decreases by 20% per decade, by 2050, there will be a potential reduction of 16.2% in dementia rates^6^.

In Brazil, an investigation identified that 50.5% of dementia cases are potentially preventable with the reduction of ten risk factors^7^. Regional differences were evaluated and some regions had higher rates of some factors than others^7^. Also, a recent publication conducted with a large Brazilian sample found that 48% of dementia cases in Brazil are attributable to 12 risk factors: less education (7.7%), hypertension (7.6%), obesity (5.6%), hearing loss (6.8%), TBI (3.1%), alcohol use (0.3%), smoking (2.1%), depression (4.4%), social isolation (0.3%), physical inactivity (4.5%), diabetes mellitus (3.1%), and air pollution (2.7%)^8^. Several percentages are above those identified in the original study^4^, resulting in great concern. In addition, the percentages for poor regions were higher than in rich regions (54.0% versus 49.0%)^8^.

Risk factors for dementia, although only identified in old age, are important throughout the life course. Longitudinal studies with middle-aged and older adults are relevant because they allow the monitoring of individual changes and the evolution of individuals during the aging process. This study aimed to compare the prevalence of risk factors for dementia in middle-aged and older adults over a two-year period and to identify what variables in baseline were predictive of cognitive decline in the follow-up assessment. The results can help monitor individual changes and the aging process, in order to prevent dementia through interventions and programs in the future.

As hypotheses, we hope to identify over a period of two years, a significant increase in the prevalence of hearing loss, high blood pressure, obesity, depression, social isolation, physical inactivity, and diabetes mellitus; a significant reduction in the prevalence of alcohol use and smoking; and the maintenance of the prevalence of education and TBI. Also, we expect that the risk factors in the baseline will predict a cognitive decline in the follow-up.

## METHODS

This is a longitudinal and quantitative study, with a follow-up evaluation after two years, conducted in the Family Health Units (FHU) in the municipality of Três Lagoas, Mato Grosso do Sul, Brazil. According to the 2010 census, the city had 101,791 inhabitants, with 16.1% aged between 45-59 years and 9.9% aged ≥60 years. In 2018, when the study began, the city had nine FHUs.

The inclusion criteria were patients aged 45 years and older, registered at a FHU in the city, and able to answer the interview questions (assessed by the interviewer’s perception). Participants were randomly selected based on a list provided by the health team.

The baseline analysis occurred between November 2018 and June 2019. The sample size was calculated using the proportion estimation formula for a finite population, with a significance level of 5% (alpha=0.05), sampling error of 6% (e=0.06), and estimate of 50% (p=0.50). Considering a finite population in the municipality of N=26,331 individuals 45 years of age and over, the minimum sample was 265 individuals, to which 10% was added to mitigate possible losses, resulting in 292 participants. During data collection, 300 patients were interviewed.

**Figure 1.**
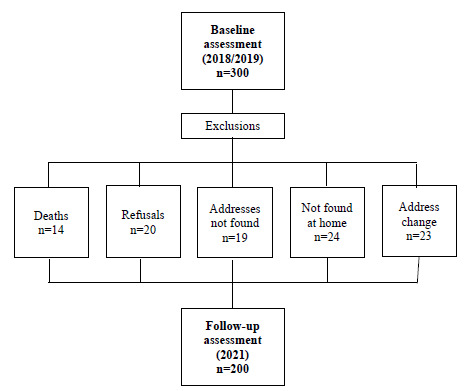
Flowchart of the initial and final number of the samples.

The follow-up assessment was conducted between February and December 2021, 2.4 years after the baseline evaluation. All baseline participants were sought to participate in the follow-up. Figure 1 describes the initial and final number of samples, as well as the excluded cases. No significant differences were identified concerning sex, age group, marital status, and education between participants who were lost in the follow-up and those reassessed, indicating a nondifferential loss (data not shown).

The evaluations were conducted by trained interviewers in the participants’ homes or the FHU premises, with an average duration of 40 min each.

The sociodemographic data collected were sex, age, marital status (with or without a partner), and personal income (up to 1 minimum wage [MW] and >1 MW).

The analyses of risk factors for dementia were based on the variables from Livingston et al.^4^. Education was evaluated in years of study (up to 4 years, over 4 years). Hearing loss was self-reported (“Do you have hearing problems that prevent you from performing any daily activities?”). TBI was evaluated by the question “Have you ever suffered a head injury (loss of consciousness >1 hour)?”. High blood pressure was self-reported (“Has a doctor ever told you that you have arterial hypertension (high blood pressure)?”). For alcohol use, in both periods, the question was “Do you currently use alcohol?”, and in the follow-up, weekly alcohol consumption was questioned and transformed into units, with consumption >21 units/week being considered as a cutoff^4^; Obesity was determined by body mass index (BMI); considering BMI≥30 kg/m^2^ for adults and BMI≥27 kg/m^2^ for older adults (≥60 years)^9^. Smoking was self-reported (“Do you currently smoke?”). Depressive symptoms were established by the Center for Epidemiological Studies - Depression (CES-D), validated in Brazil, in which the final score varies from 0 (zero) to 60 points (higher scores, higher depressive symptoms), and the cutoff score for the presence of depressive symptoms for adults was ≥16 points, and for older adults, ≥12 points^10^ ^,^ ^11^. Social isolation, in the baseline, was assessed by the question “Do you feel socially isolated?” and in the follow-up, question 15 of the UCLA-BR Loneliness Scale^12^ was used: “I feel isolated from other people”, with the answers “sometimes” and “frequently” categorized as “yes”. Physical inactivity was identified using the International Physical Activity Questionnaire (IPAQ) short version, validated in Brazil^13^ ^,^ ^14^. The participants were classified as active (at least 150 min of moderate activity and/or 75 min of vigorous activity per week) or inactive (did not reach the minimum time). Diabetes mellitus was self-reported (“Has a doctor ever told you that you have diabetes?”).

The Mini-Mental State Examination (MMSE) was applied, which is a cognitive status screening instrument with a maximum score of 30 points^15^ ^,^ ^16^. We used the previously proposed cutoff scores to classify the participants into with cognitive decline and without cognitive decline according to their education (13 for illiterate, 18 for 1-7 years of schooling, and 26 for 8 years and over)^16^.

The data were analyzed in Statistical Package for Social Sciences (SPSS) version 25.0. Descriptive analyses are presented in a table format with frequency (percentage), mean, and standard deviation (SD). The prevalence of each risk factor and cognitive performance was calculated with respective 95% confidence interval (95%CI) in the baseline and follow-up assessments. Prevalence data were compared using the McNemar’s test. To identify which risk factors for dementia in baseline were predictive of cognitive decline in the follow-up assessment, we divided the dependent variable cognitive performance into three categories:

- Remained unchanged or improved the cognitive performance;
- Remained with cognitive decline; and
- Started to have cognitive decline. Individual multinomial logistic regression models were performed.

The first group was considered as the reference in regression models. All baseline risk factors were independent variables. Sex, age, and education were used as confounding factors. Results with a p-value (p)≤0.050 were considered significant.

The study was approved by the Human Research Ethics Committee of the Federal University of Mato Grosso do Sul (opinions numbers 2,596,194 and 4,467,405). All participants read and signed the informed consent form before the two interviews.

## RESULTS

Table 1 presents the characterization data of the 200 participants in the baseline assessment and the comparison of risk factors for dementia and participants’ cognitive performance between the two periods. The prevalence of low education, TBI, and smoking remained the same. The risk factors that showed significant changes were high blood pressure (p=0.016), physical inactivity (p<0.001) with an increased prevalence, and social isolation (p=0.032) with a reduced prevalence. Also, cognitive performance was statistically different (p=0.047), with a higher prevalence of individuals with cognitive decline in the follow-up assessment.

**Table 1.** Characterization of participants (baseline) and comparison of risk factors for dementia and participants’ cognitive performance between the two assessments (n=200).

Variable	Baseline (2018/2019)	Follow-up (2021)	p-value*
Sex (female, %)	66.5	66.5	
Age (mean, SD)	61.7 (11.0)		
Marital status (with partner, %)	56.0	56.0	
Personal income (up to 1 MW, %)	50.0	50.0	
Education (mean, SD)	5.8 (4.4)	5.8 (4.8)	-
up to 4 years	44.0 (37.3-50.9)	44.0 (37.3-50.9)	-
Self-reported hearing loss (yes)	15.5 (11.1-21.2)	18.0 (13.3-23.9)	0.442
Traumatic brain injury	10.5 (7.0-15.5)	10.5 (7.0-15.5)	-
High blood pressure (yes)	55.0 (48.1-61.7)	62.0 (55.1-68.4)	**0.016**
Alcohol use (yes)	23.5 (18.2-29.8)	25.5 (20.0-32.0)	0.557
Alcohol use (>21 units/week)	-	5.5 (3.1-9.6)	
Obesity (yes)	66.0 (59.2-72.2)	61.4 (54.6-68.0)	0.229
Smoking (yes)	19.0 (14.2-25.0)	19.0 (14.2-25.0)	-
Depressive symptoms (yes)	55.0 (48.1-61.7)	52.0 (45.1-58.8)	0.539
Social isolation (yes)	25.5 (20.0-32.0)	18.0 (13.3-23.9)	**0.032**
Physical inactivity	58.5 (51.6-65.1)	74.5 (68.0-80.0)	**<0.001**
Self-reported diabetes mellitus (yes)	30.0 (24.1-36.7)	33.5 (27.3-40.3)	0.092
Cognitive performance (with cognitive decline)	18.0	24.5	**0.047**

Abbreviations: SD, standard deviation; MW, minimum wage.

Notes: *McNemar’s test. Variables were presented in 95% confidence interval, except where indicated. Bold indicates p-values<0.05.

Factors associated with longitudinal cognitive performance after two years are presented in Table 2. No risk factors were associated with remaining cognitive decline after two years. Participants with depressive symptoms were at higher risk of having cognitive decline after two years (odds ratio [OR]=3.43, 95%CI 1.26-9.38, p=0.016).

**Table 2.** Regression models to identify risk factors for dementia as factors associated with longitudinal monitoring of cognitive performance.

Variable	OR	95%CI	p-value
Remained with cognitive decline			
Education	1.10	0.98-1.23	0.102
Self-reported hearing loss	3.02	0.98-9.31	0.055
Traumatic brain injury	1.83	0.52-6.48	0.352
High blood pressure	0.71	0.27-1.86	0.491
Alcohol use	1.11	0.39-3.16	0.845
Obesity	1.05	0.39-2.79	0.928
Smoking	0.88	0.27-2.90	0.833
Depressive symptoms	1.49	0.59-3.75	0.395
Social isolation	1.74	0.65-4.65	0.271
Physical inactivity	1.81	0.70-4.72	0.222
Self-reported diabetes mellitus	1.56	0.58-4.22	0.382
Started to have cognitive decline			
Education*	1.08	0.97-1.21	0.183
Self-reported hearing loss	1.98	0.64-6.17	0.239
Traumatic brain injury	1.29	0.33-4.99	0.718
High blood pressure	0.63	0.25-1.59	0.326
Alcohol use	1.33	0.50-3.55	0.566
Obesity	0.81	0.33-2.01	0.653
Smoking	1.11	0.37-3.31	0.854
Depressive symptoms	3.43	1.26-9.38	**0.016**
Social isolation	2.42	0.96-6.11	0.060
Physical inactivity	1.24	0.51-3.01	0.628
Self-reported diabetes mellitus	0.80	0.29-2.18	0.657

Abbreviations: OR, odds ratio; CI, confidence interval.

Notes: *Education was adjusted for sex and age. All other variables were adjusted for sex, age, and education. Bold indicates p-values<0.05.

## DISCUSSION

By comparing the modifiable risk factors for dementia in a two-year follow-up, we found that the prevalence of high blood pressure and physical inactivity increased, and social isolation decreased. For low education, TBI, and smoking, the frequency was maintained. The prevalence of individuals with cognitive decline in the follow-up was significantly higher than in baseline. Depressive symptoms predicted cognitive decline after two years.

An increase in the prevalence of high blood pressure was expected because of the increase in the age of participants throughout the study. The global rate of hypertension in 2012 was 31.1%^17^. In Brazil, the prevalence of hypertension in individuals over 18 years of age was 19.9% in 2008, 21.3% in 2013, and 23.9% in 2019^18^. Other investigations point to an increase in the prevalence of hypertension among Brazilians over the years, as well as its increase as age groups increase^18^ ^,^ ^19^ ^,^ ^20^. A systematic review and meta-analysis of 209 prospective studies evaluated the relationship between blood pressure and the risk of cognitive disorders and dementia. There are indications that in middle age, high systolic and diastolic blood pressures values (i.e., ≥140 mmHg and ≥80 mmHg, respectively) are related to a higher risk for dementia and Alzheimer’s disease^21^. However, the association between altered blood pressure and changes in cognition may not be the same in older adults, as research results did not indicate strong evidence^21^. A cohort study identified that the higher the blood pressure, the lower the risk of dementia in older age groups^22^. It is worth pointing out a caveat in the association, which consists of the existence of other mechanisms that can influence the occurrence of dementia, such as the use of antihypertensives, sample selection, and chronicity of the disease.

Most hypertensive Brazilians (74.7%) received assistance in 2018, and older adults were those who most frequently reported health care for the disease. However, the Central-West region had a lower percentage of this assistance (68.5%)^18^. Considering that the present study was conducted in this region, it is concluded that this region needs greater investment in healthcare for hypertension, which may reflect the prevalence of dementia in the future.

Physical inactivity also showed an increased prevalence in the present study, according to our initial hypothesis, particularly because of the circumstances imposed by the COVID-19 pandemic. A study conducted with adults compared the practice of physical activity before and at the beginning of the pandemic and found a reduction in the levels^23^. Physical activity was assessed in older adults four times: January, April, and August 2020 and January 2021. The time spent practicing physical activity was reduced by 33.3% in the first reassessment, 28.3% in the second, and 40% in the third reassessment compared with January 2020^24^.

A meta-analysis of cohort studies found that high levels of physical activity were associated with a linear reduction in the onset of dementia^25^. In addition to prevention, exercise also helps to better manage the disease, as individuals with mild cognitive impairment have demonstrated improvements in global cognition, executive functioning, attention, and memory with increased physical activity^26^.

A significant reduction in the prevalence of social isolation was identified between the two study periods. An investigation conducted with adults and older adults during the pandemic showed that older participants reported a feeling of less social isolation than younger people, indicating that their routines may have been less affected or that they are more resilient than younger people^27^. Furthermore, it can be inferred that with the restrictions imposed by the pandemic, older adults’ family members were more present at home than before the pandemic (home office and remote schooling), which may have resulted in a lower perception of isolation. A cohort study with 462,619 participants indicated that social isolation was associated with a greater risk of dementia. In cases of social isolation, individuals have lower gray matter volumes in the temporal, frontal, hippocampal, and other regions, predisposing them to dementia^28^. Therefore, reducing social isolation may be a beneficial factor in reducing the prevalence of dementia in the future.

The only predictor of cognitive decline in follow-up assessment was depressive symptoms. A meta-analysis of longitudinal studies found that patients living with depression are at higher risk of dementia than the general population^29^. In addition, a systematic review found that dementia and depressive symptoms are related, but the nature of the relationship is inconclusive because they can have common risk factors; depressive symptoms can be a prodromal symptom or a risk factor for dementia^30^. Even considering that there are inconclusive mechanisms in this relationship, depressive symptoms are an important factor to be considered in interventions to prevent cognitive decline and dementia. In Brazil, this factor was equally significant either in the rich and poor regions of the country^8^.

The present study has limitations:

- The sample loss between the two data collections, despite being nondifferential, impacted the size of the follow-up sample;
- The data cannot be generalized as they only represent a small sample of a Brazilian municipality;
- Although we applied instruments validated to Brazilian culture to assess risk factors, some were self-reported (e.g. hearing loss, diabetes, hypertension), therefore subject to participants’ memory bias, and so, future studies that address the medical diagnosis of these conditions are recommended;
- Even though we considered the pandemic context in our discussion, the follow-up assessment took place in 2021, when the country still had high incidence and mortality rates from COVID-19, which may have interfered with the participants’ responses.

Despite the limitations, the data found contribute to the literature regarding risk factors for dementia in middle-aged and older adults, as it includes individuals aged 45 years and over, making it possible to analyze the aging process and enable early interventions.

The most prevalent risk factors for dementia in the study sample were physical inactivity, high blood pressure, obesity, and depressive symptoms. High blood pressure and physical inactivity showed a significant increase in prevalence over a two-year period; social isolation showed a significant reduction; and low education, TBI, and smoking remained the same. An increase in the prevalence of cognitive decline was observed in the period. Depressive symptoms predicted cognitive decline after two years.

We confirmed the previously raised hypotheses of an increase in the prevalence of high blood pressure and physical inactivity and the maintenance of the prevalence of low education and TBI. We partially confirmed the hypothesis that the risk factors at baseline would predict a cognitive decline in the follow-up assessment because only depressive symptoms were significant. The other hypotheses were not confirmed. It is recommended that new studies be conducted to monitor risk factors for dementia in other samples and to analyze these factors in the post-pandemic period.

The research results can help in the development of interventions, especially in Brazil because there are few public policies aimed at addressing the growing demand for dementia. In this context, early assessment and health monitoring of the aging population are essential for better disease management and prevention.

## References

[1] World Health Organization (2021). Global status report on the public health response to dementia.

[2] Ranson JM, Rittman T, Hayat S, Brayne C, Jessen F, Blennow K (2021). Modifiable risk factors for dementia and dementia risk profiling. A user manual for Brain Health Services--part 2 of 6. Alzheimers Res Ther.

[3] Livingston G, Sommerlad A, Orgeta V, Costafreda SG, Huntley J, Ames D (2017). Dementia prevention, intervention, and care. Lancet.

[4] Livingston G, Huntley J, Sommerlad A, Ames D, Ballard C, Banerjee S (2020). Dementia prevention, intervention, and care: 2020 report of the Lancet Commission. Lancet.

[5] Mukadam N, Sommerlad A, Huntley J, Livingston G (2019). Population attributable fractions for risk factors for dementia in low-income and middle-income countries: an analysis using cross-sectional survey data. Lancet Glob Health.

[6] Oliveira D, Otuyama LJ, Mabunda D, Mandlate F, Gonçalves-Pereira M, Xavier M (2019). Reducing the number of people with dementia through primary prevention in Mozambique, Brazil, and Portugal: an analysis of population-based data. J Alzheimers Dis.

[7] Borelli WV, Leotti VB, Strelow MZ, Chaves MLF, Castilhos RM (2022). Preventable risk factors of dementia: Population attributable fractions in a Brazilian population-based study. Lancet Reg Health Am.

[8] Suemoto CK, Mukadam N, Brucki SMD, Caramelli P, Nitrini R, Laks J (2023). Risk factors for dementia in Brazil: differences by region and race. Alzheimers Dement.

[9] Brasil. Ministério da Saúde. Secretaria de Atenção à Saúde. Departamento de Atenção Básica (2011). Orientações para a coleta e análise de dados antropométricos em serviços de saúde.

[10] Silveira DX, Jorge MR (1998). Propriedades psicométricas da escala de Rastreamento Populacional para Depressão CES-D em populações clínicas e não-clínicas de adolescentes e adultos jovens. Rev Psiquiatr Clín.

[11] Batistoni SST, Néri AL, Cupertino AP (2010). Validade e confiabilidade da versão Brasileira da Center for Epidemiological Scale - Depression (CES-D) em idosos Brasileiros. Psico-USF.

[12] Barroso SM, Andrade VS, Oliveira NR (2016). Escala Brasileira de Solidão: análises de resposta ao item e definição dos pontos de corte. J Bras Psiquiatr.

[13] Matsudo S, Araújo T, Marsudo V, Andrade D, Andrade E, Oliveira LC (2001). Questionário Internacional de Atividade Física (IPAQ): estudo de validade e reprodutibilidade no Brasil. Rev Bras Ativ Fís Saúde.

[14] Benedetti TRB, Antunes PC, Rodriguez-Añez CR, Mazo GZ, Petroski EL (2007). Reprodutibilidade e validade do Questionário Internacional de Atividade Física (IPAQ) em homens idosos. Rev Bras Med Esporte.

[15] Folstein MF, Folstein SE, McHugh PR (1975). “Mini-mental state”. A practical method for grading the cognitive state of patients for the clinician. J Psychiatr Res.

[16] Bertolucci PHF, Brucki SMD, Campacci SR, Juliano Y (1994). O Mini-Exame do Estado Mental em uma população geral: impacto da escolaridade. Arq Neuropsiquiatr.

[17] Mills KT, Bundy JD, Kelly TN, Reed JE, Kearney PM, Reynolds K (2016). Global Disparities of Hypertension Prevalence and Control: A Systematic Analysis of Population-Based Studies From 90 Countries. Circulation.

[18] Julião NA, Souza A, Guimarães RRM (2021). Tendências na prevalência de hipertensão arterial sistêmica e na utilização de serviços de saúde no Brasil ao longo de uma década (2008-2019). Ciênc Saúde Coletiva.

[19] Lobo LAC, Canuto R, Dias-da-Costa JS, Pattussi MP (2017). Tendência temporal da prevalência de hipertensão arterial sistêmica no Brasil. Cad Saúde Pública.

[20] Lima-Costa MF, Andrade FB, Souza PRB, Neri AL, Duarte YAO, Castro-Costa E (2018). The Brazilian Longitudinal Study of Aging (ELSI-Brazil): objectives and design. Am J Epidemiol.

[21] Ou YN, Tan CC, Shen XN, Xu W, Hou XH, Dong Q (2020). Blood Pressure and Risks of Cognitive Impairment and Dementia: A Systematic Review and Meta-Analysis of 209 Prospective Studies. Hypertension.

[22] van Dalen JW, Brayne C, Crane PK, Fratiglioni L, Larson EB, Lobo A (2022). Association of systolic blood pressure with dementia risk and the role of age, u-shaped associations, and mortality. JAMA Intern Med.

[23] Puccinelli PJ, Costa TS, Seffrin A, Lira CAB, Vancini RL, Nikolaidis PT (2021). Reduced level of physical activity during COVID-19 pandemic is associated with depression and anxiety levels: an internet-based survey. BMC Public Health.

[24] Yamada M, Kimura Y, Ishiyama D, Otobe Y, Suzuki M, Koyama S (2021). The influence of the COVID-19 pandemic on physical activity and new incidence of frailty among initially non-frail older adults in Japan: a follow-up online survey. J Nutr Health Aging.

[25] Dominguez LJ, Veronese N, Vernuccio L, Catanese G, Inzerillo F, Salemi G (2021). Nutrition, physical activity, and other lifestyle factors in the prevention of cognitive decline and dementia. Nutrients.

[26] Nuzum H, Stickel A, Corona M, Zeller M, Melrose RJ, Wilkins SS (2020). Potential benefits of physical activity in MCI and dementia. Behav Neurol.

[27] Birditt KS, Turkelson A, Fingerman KL, Polenick CA, Oya A (2021). Age differences in stress, life changes, and social ties during the COVID-19 pandemic: implications for psychological well-being. Gerontologist.

[28] Shen C, Rolls ET, Cheng W, Kang J, Dong G, Xie C (2022). Associations of social isolation and loneliness with later dementia. Neurology.

[29] Chan JYC, Yiu KKL, Kwok TCY, Wong SYS, Tsoi KKF (2019). Depression and antidepressants as potential risk factors in dementia: a systematic review and meta-analysis of 18 longitudinal studies. J Am Med Dir Assoc.

[30] Wiels W, Baeken C, Engelborghs S (2020). Depressive symptoms in the elderly-an early symptom of dementia? A systematic review. Front Pharmacol.

